# Development, characterization, and applications of multi-material stereolithography bioprinting

**DOI:** 10.1038/s41598-021-82102-w

**Published:** 2021-02-04

**Authors:** Bagrat Grigoryan, Daniel W. Sazer, Amanda Avila, Jacob L. Albritton, Aparna Padhye, Anderson H. Ta, Paul T. Greenfield, Don L. Gibbons, Jordan S. Miller

**Affiliations:** 1 Department of Bioengineering, Rice University, Houston, TX USA; 2 Department of Thoracic/Head and Neck Medical Oncology, The University of Texas MD Anderson Cancer Center, Houston, TX USA; 3 Department of Molecular and Cellular Oncology, The University of Texas MD Anderson Cancer Center, Houston, TX USA

**Keywords:** Biomedical engineering, Biomaterials, Gels and hydrogels

## Abstract

As a 3D bioprinting technique, hydrogel stereolithography has historically been limited in its ability to capture the spatial heterogeneity that permeates mammalian tissues and dictates structure–function relationships. This limitation stems directly from the difficulty of preventing unwanted material mixing when switching between different liquid bioinks. Accordingly, we present the development, characterization, and application of a multi-material stereolithography bioprinter that provides controlled material selection, yields precise regional feature alignment, and minimizes bioink mixing. Fluorescent tracers were first used to highlight the broad design freedoms afforded by this fabrication strategy, complemented by morphometric image analysis to validate architectural fidelity. To evaluate the bioactivity of printed gels, 344SQ lung adenocarcinoma cells were printed in a 3D core/shell architecture. These cells exhibited native phenotypic behavior as evidenced by apparent proliferation and formation of spherical multicellular aggregates. Cells were also printed as pre-formed multicellular aggregates, which appropriately developed invasive protrusions in response to hTGF-β1. Finally, we constructed a simplified model of intratumoral heterogeneity with two separate sub-populations of 344SQ cells, which together grew over 14 days to form a dense regional interface. Together, these studies highlight the potential of multi-material stereolithography to probe heterotypic interactions between distinct cell types in tissue-specific microenvironments.

## Introduction

Mammalian organ function relies on controlled interactions between discrete tissue domains that are physically separate yet biochemically linked. These interfaces are exemplified by vascular capillaries, with which parenchymal and stromal tissues exchange gases, small molecules, and proteins, without transferring larger components such as albumin, fibrinogen, or red and white blood cells. The development and progression of many diseases are often marked by disruptions of these physical boundaries, which regulate the heterotypic dynamics between unique cell populations and extra-cellular components. For example, macular degeneration often begins when retinal blood vessels penetrate through the thin pigment epithelium^1^, and cancer metastasis is largely contingent on tumor cell invasion of the surrounding healthy tissues^2^. To better understand the heterotypic interactions that drive these spatial aberrations without the need for costly and variable animal models, researchers must be able to fabricate biological structures with exquisite control over spatial heterogeneity throughout all three dimensions.

Sequentially casting distinct combinations of cells and hydrogels into physical molds is the simplest method to achieve spatial heterogeneity. By stacking layers of unique compositions, zonal organization with discrete layers and gradient transitions can be achieved^3^. However, there exist tradeoffs between complexity and precision, and these stratified constructs are often limited to uniaxial control. The need for biaxial heterogeneity can be addressed with inkjet printing^4,5^, microcontact printing^6^, or photolithography^7^, but these strategies do not easily scale to full 3D control.

Multi-nozzle extrusion printing currently leads the field in heterogeneous biofabrication^8–10^, with liquid or shear-thinning bioinks that rapidly solidify as they’re pushed through a circular nozzle, allowing sequential material deposition without risk of material mixing. However, line-by-line deposition of cylindrical filaments can lead to junctional seams and voids that diminish architectural fidelity and may even lead to structural deformation of softer materials during longitudinal cell culture^11^. Newer approaches within the extrusion framework have used switching Y-junction nozzles to produce monolithic filaments with continuously tunable composition, but the printed structures still suffer from issues of filament interdigitation^9^. Ultimately, these regional fabrication artifacts may confound interpretations of cellular migration patterns and contact-mediated phenomena, which are often of interest in microphysiologic systems.

Compared to multi-nozzle extrusion bioprinting, 3D printing with stereolithography (SLA) does not require physical alignment and fusion of adjacent filaments. Whereas extrusion printers physically deposit cylindrical filaments line-by-line, stereolithography relies on spatially controlled illumination to selectively crosslink liquid bioink into solid hydrogel features. This layer-by-layer printing strategy therefore offers an exciting opportunity to explore multi-material integration with fewer concerns over unintended voids or seams between material domains. SLA also provides access to architectures with over-hanging features such as hollow vessels and free-standing arches^12,13^, which would otherwise be difficult to achieve with traditional methods of biofabrication.

Throughout the SLA printing process, non-illuminated regions remain liquid, leaving behind an excess volume of uncrosslinked bioink that sticks to and coats nascent structures during printing. Therefore, switching the active structure between different materials invariably compromises cellular and biochemical purity. To limit mixing, several strategies have previously been explored. Typically, the active structure is lifted out of the liquid polymer solution and washed with water or saline before returning to the build area. This cleaning step is simple and effective even when performed as a manual process^14,15^, although automated fluid-exchanging manifolds^16,17^ and rotating material baths^18^ have also been developed which help reduce fabrication times. A separate strategy is to clear off residual liquid polymer with compressed air^19^, although this technique was originally developed for denser plastic materials and is likely incompatible with cellularized, hydrogel bioinks, which must be well hydrated at all times to maintain cell viability. While these strategies are exciting and effective, the lack of commercial or open-source hardware has likely slowed adoption by the wider bioprinting community. Furthermore, the architectural complexity, print fidelity, and biological applications enabled by these techniques have yet to be fully explored.

To address the need for spatially controlled biological structures, we developed a multi-material stereolithography (MMSLA) bioprinter that uses an automated material selection process and manual saline rinsing step to form heterogeneous structures without unwanted mixing. Using this semi-automated printer, we first demonstrated monolithic integration of multiple synthetic and naturally derived bioinks. Next, printing of fluorescent tracers and morphometric image analysis confirmed high geometric fidelity within an architecturally rich design space. To explore potential biological applications enabled by this system, we used a soft GelMA bioink to print 3D hydrogel environments with discrete cellular and acellular domains. When encapsulated in multi-material core/shell architectures, we found that populations of dispersed cancer cells underwent considerable proliferation and that pre-formed multicellular aggregates exhibited phenotypically appropriate invasive morphologies in response to hTGF-β1. Finally, we highlight the potential of our system to interrogate heterotypic interactions by constructing a simplified model of intratumoral heterogeneity, which grew over 14 days to form a dense mosaic with seamless regional interfaces suitable for future studies of contact-mediated tumor evolution.

## Results

### Development of a multi-material bioprinter for hydrogel stereolithography

SLA is a light-based 3D printing technique that can be used to fabricate both cellular and acellular structures. Compared to traditional extrusion printing, where cylindrical filaments of liquid or shear-thinning bioink are physically deposited by a moving nozzle, SLA does not require physical contact to manipulate individual layers and features. Instead, SLA uses controlled illumination to selectively photocrosslink liquid bioinks into solid features, offering higher spatial resolution than extrusion printers by using precision lasers or video projectors. But commercial SLA systems are almost exclusively designed to print hard, plastic materials for non-biological applications and do not offer multi-material capability. The difficulty of modifying proprietary hardware/software to accommodate soft biological materials—such as hydrogels—has impeded widespread adoption by the bioprinting community.

**Figure 1:**
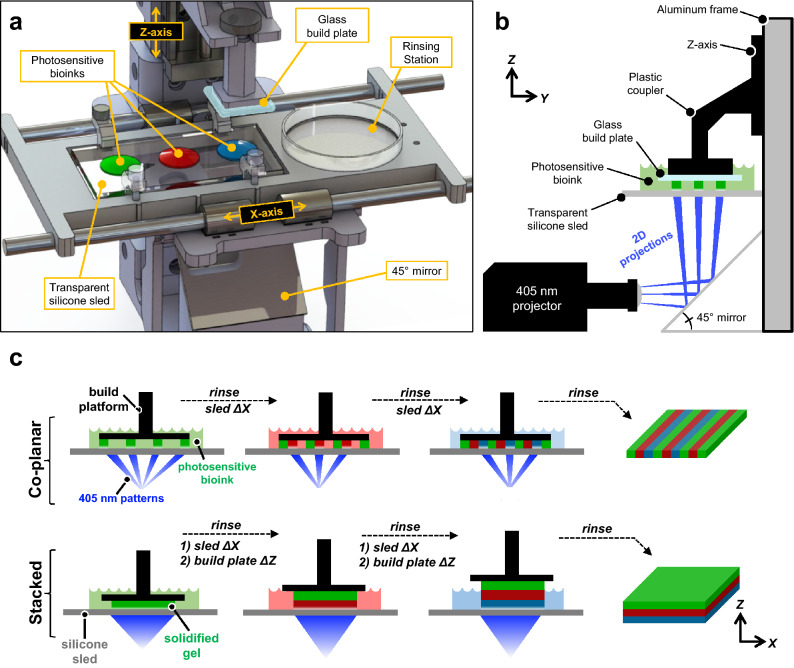
Design and workflow for a multi-material stereolithography bioprinter. (**a**) Perspective view rendering of bioprinter components and final design; note the presence of a motorized silicone sled, which translates laterally in the X-axis to automate bioink selection and rinsing. (**b**) Side view schematic showing the glass build plate lowered into a bioink droplet, creating a thin first layer (50 μm) for photocrosslinking. (**c**) Print workflow for fabricating monolithic gels with spatially controlled heterogeneity. The motorized sled allows nascent structures to interface with separate bioinks of variable chemical or cellular composition, providing heterogeneity within individuals layer (co-planar; XY) and across sequential layers (stacked; Z).

To address this gap in technology, we have developed a fully open-source MMSLA bioprinter consisting of inexpensive, readily available components that allow for multi-material fabrication of spatially heterogeneous hydrogel structures (Fig. 1a). Because the theoretical resolution for any SLA system is primarily limited by its optical source, we began development by testing two commercially available projection sources: a classroom-style video projector (38 µm pixels, broad-spectrum white light lamp) and a production-ready industrial projector, specifically built for 3D printing applications (50 µm pixels, 405 nm LED). Both projection sources were used without any modification. While the classroom projector had marginally smaller pixels—and therefore higher theoretical resolution—the industrial projector allowed for shorter fabrication times due to its greater output light intensity in the 405 nm absorbance range of our bioink photoinitiator (lithium phenyl-2,4,6-trimethylbenzoylphosphinate; LAP). Furthermore, the light intensity profile of the classroom projector had a noticeable gaussian distribution, while the industrial projector displayed greater homogeneity and was therefore used for all quantitative and cellular studies.

To begin each print, a stepper motor-driven linear actuator (Z-axis) lowers a glass build plate into the first bioink droplet (100–1000 μL). This creates a thin layer (50 μm) between the build plate and an underlying transparent silicone sled (PDMS-coated glass), which as designed can hold up to 4 different bioinks. This thin initial layer is then selectively photocrosslinked into a soft yet solid hydrogel feature using a 2D grayscale light pattern (i.e. photomask), which is generated by the projector and directed up through the transparent sled using a 45° mirror (Fig. 1b). The crosslinked hydrogel features preferentially adhere to the hydrophilic glass build plate, rather than the hydrophobic silicone sled, allowing the build plate to serve as a moving substrate for the growing object. After each exposure, the build plate raises up by a user-specified layer height (50–300 μm) drawing more liquid under the build plate to be photocrosslinked by the next photomask. Sequential features covalently laminate, and the process repeats layer-by-layer in the Z-axis until the entire 3D object is created.

Critically, multi-material capability is facilitated by a second linear actuator (X-axis) that drives the silicone sled laterally between bioink droplets to automate material selection. These motions are digitally synchronized with the build plate (Z-axis) and photomask projection sequence (Fig. 1c), enabling heterogeneous bioink integration within individual layers (co-planar) and across successive layers (stacked). When switching between materials, the Z-axis first lifts the build plate and active structure up and out of the current bioink droplet. Then, the X-axis sled moves laterally to bring a small, empty petri dish under the build plate so that spent rinsing fluid can be captured during the following step. Here, a syringe and long 20 GA needle are manually used to quickly expel a small volume (2–5 mL) of phosphate buffered saline (PBS) directly onto the build plate and structure. Next, an autoclaved cellulose lab wiper (Kimwipe) is used to wick away any residual saline from the build plate so that the next bioink droplets do not become diluted. Together, the X- and Z-axes then re-position the build plate over and into the next bioink droplet so that printing can continue.

Intriguingly, we found that the active fluid flow associated with this rinsing technique was essential in cleansing the hydrogel structures and maintaining bioink purity. We observed that merely submerging the gel and build plate in a saline bath was insufficient to fully cleanse the hydrogel object of excess liquid, resulting in unwanted mixing. Additionally, we found that structures rinsed with PBS alone did not always laminate with the subsequent material, while the inclusion of dilute photopolymer (2.5–10 wt%) in the PBS saline provided more consistent lamination. The photopolymer itself was chosen to match the subsequent bioink (PEGDA, GelMA, etc.), thus maintaining consistent biochemical purity.

In summary, the incorporation of an X-axis sled provides a subtle yet valuable improvement over traditional SLA approaches, offering access to multi-material structures with simple and open-source designs. In its current form, we cautiously yet enthusiastically classify this printer as a semi-automated system. While the two motors and projector are digitally synchronized at efficient speeds (print times ≤ 15 min), further innovation is still needed to integrate the manual saline rinse step.

### Construction of multi-material hydrogels with broad design freedoms

**Figure 2:**
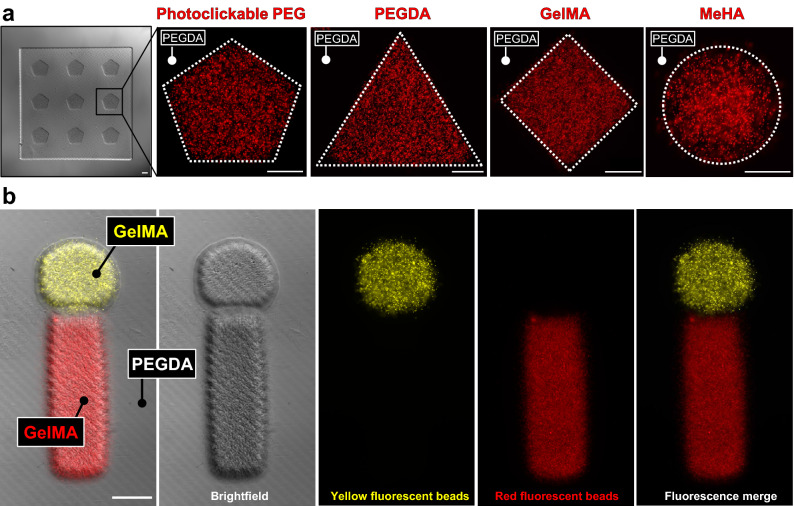
Integration of multiple bioinks. (**a**) Multi-material stereolithography is compatible with a range of bioinks, including synthetic polymers (photoclickable PEG, PEGDA) and naturally derived polymers (MeHA, GelMA). In each example, a geometrically arrayed feature layer with encapsulated fluorescent beads was printed atop a square PEGDA base. (**b**) Monolithic integration of 3 bioinks, highlighting successful spatial segmentation with few if any beads off-target. All scale bars = 250 μm.

To demonstrate the flexibility of hydrogel bioinks compatible with our printer, we first built structures using a variety of synthetic and naturally derived hydrogel materials: poly(ethylene glycol) diacrylate (PEGDA), methacrylated hyaluronic acid (MeHA), gelatin methacrylate (GelMA), and photoclickable PEG (norbornene- and thiol-modified) (Fig. 2a). Rather than simply printing hydrogel features directly onto a glass substrate—which is otherwise easily achievable through traditional photolithography—here we construct heterogeneity in the Z-direction by covalently stacking sequential materials: a non-fluorescent PEGDA base was printed first, followed by a geometrically arrayed feature layer of variable chemistry. This approach was then extended by including a second fluorescent material to the feature layer, providing a means to visually assess whether our rinsing strategy was successful in preventing bioink mixing. Gels with red and yellow fluorescent beads were printed in a post/stem geometry (Fig. 2b), with resulting structures containing no beads present outside their intended domains. This ability to cleanly partition features within a larger multi-material pattern is likely critical for biological experiments, as misplaced cells could obscure the effects and interpretations of heterotypic interfacial behaviors.

**Figure 3:**
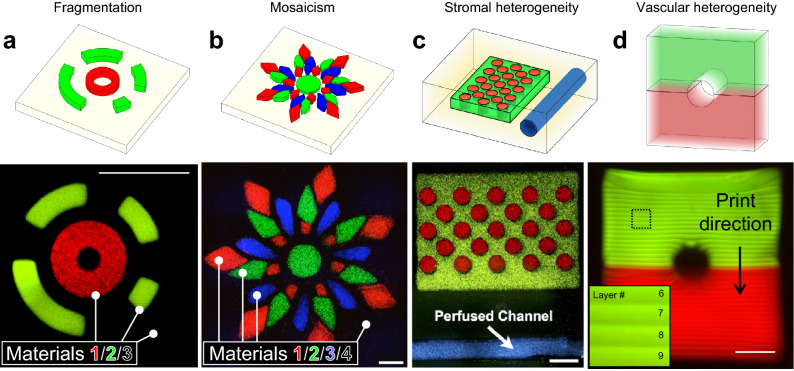
Multi-material printing of heterogeneous structures with broad design freedoms. (**a**) Annular hydrogel features with encapsulated fluorescent beads, printed atop a non-fluorescent base, yielding a monolithic 3-material structure with discrete, fragmented features at meso scale resolution (≈250 μm). (**b**) A 4-material floral mosaic. (**c**) A thick gel with stromal heterogeneity, featuring a hollow vessel that supported perfusion with blue fluorescent beads. (**d**) Side view of a two-component vessel (fluorescent dextrans encapsulated); inset highlights void-free covalent lamination between print layers. All scale bars = 1000 μm.

We then sought to more comprehensively explore the broad geometric design space afforded by our printer’s multi-material control. Fluorescent beads were used to formulate 4 separate bioinks (PEGDA; 3.4 kDa, 15 wt%), with which a collection of architectural motifs was explored: fragmentation of discrete features, mosaic assembly, stromal heterogeneity, and vascular heterogeneity (Fig. 3a-d). Excitingly, over 30 layers were needed to accommodate the two-component vessel shown in Fig. 3d. Together, these findings implicate hydrogel MMSLA as a powerful tool for generating previously unexplored hydrogel architectures that could be useful for tissue engineers and fundamental biologists.

### Morphometric image analysis of multi-material print fidelity

**Figure 4:**
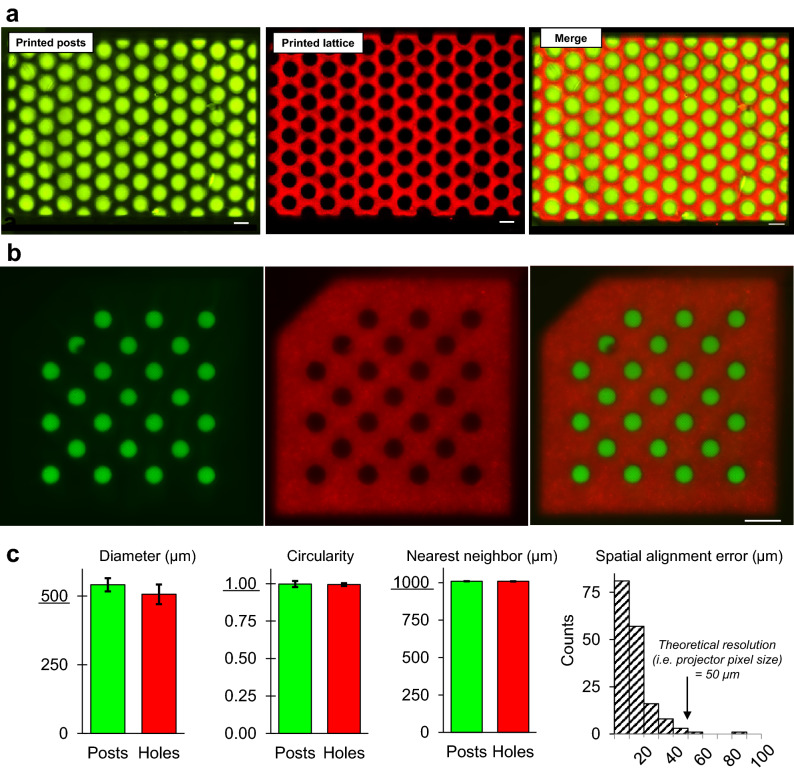
Morphometric quantification of multi-material gels highlights reproducible architectural fidelity. (**a**) Multi-material checkerboard architecture with fluorescent dextrans encapsulated; posts were tightly nested within holes of an open lattice, yielding a covalently bonded monolithic structure free of seams, voids, or other unwanted interfacial defects. (**b**) A similar checkerboard (20% PEGDA, 6 kDa) used as a test case for (**c**) morphometric image analysis. With respect to dimensions from the original CAD file, it was observed that printed feature diameters (500 μm), circularities (1.00), and nearest neighbor distances (1000 μm) were preserved. Importantly, spatial alignment errors for > 98% of nested post/hole pairs were smaller than our projector’s theoretical resolution (i.e. pixel size). This suggests performance was limited by optical hardware, rather than hydrogel formulation or photochemical print parameters. For all graphs n = 3 batches of print solution. Scale bars = 1000 μm.

For our multi-material printer to be useful in studies of heterotypic cell interactions, it must be able to faithfully reproduce multi-material design files. We were specifically interested in testing the print fidelity of high-density checkerboard patterns (Fig. 4a), which provide a large number of interfacial edges to study cell dynamics at higher experimental throughputs. To that end, we quantified morphometric parameters associated with PEGDA hydrogels consisting of hexagonally packed posts (Ø = 500 μm) tightly nested within holes of an open lattice (Fig. 4b). Bioinks for the post and lattice features were mixed with fluorescently labeled dextrans of high enough molecular weight (70 kDa, 150 kDa) to be physically immobilized within the hydrogel’s polymer network, thus enabling quantitative image analysis. Using a custom MATLAB image processing script, we confirmed that all features maintained precise and accurate diameters, circularities, and nearest neighbor distances (Fig. 4c). Excitingly, we also found that > 98% of the spatial alignment errors between centroids of nested post/hole pairs were < 50 µm, which represents < 10% deviation from the original design and is within the theoretical resolution limit of the projector’s pixel size (50 μm). Together, these findings demonstrate a high degree of geometric fidelity within the meso-scale regime (250 < Ø < 1000 μm), which is a length scale often of interest in microphysiological systems.

### Bioprinted architectures with controlled spatial heterogeneity preserve functional activity of single cells and multicellular aggregates

**Figure 5:**
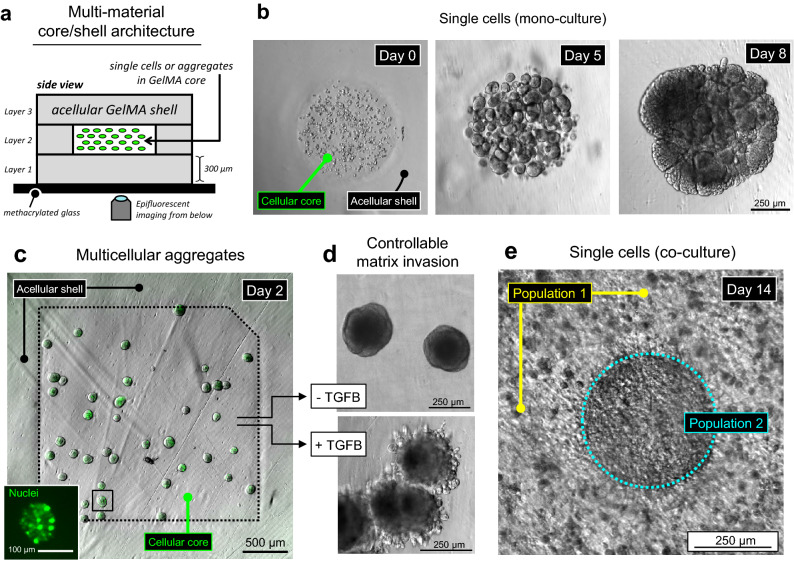
Growth and functional response of bioprinted cells and multicellular aggregates. (**a**) Schematized core/shell architecture of multi-material bioprinted constructs (5 wt% GelMA), viewed from the side. The surrounding a cellular shell provides a large growth volume for 3D cell migration, preventing unwanted 2D cell spreading along the gel’s exterior surfaces. (**b**) Bioprinting of single 344SQ cells within sharply defined cylindrical cores. Due to the saline rinsing step between material selections, the surrounding a cellular shell is completely free of off-target cells. Over 5 days in culture, the encapsulated cells extensively proliferated and self-assembled into spheroids, a well-known behavior of 344SQ cells in other naturally derived hydrogel materials. By day 8, spheroids further coalesced into a dense unified structure (n = 3 gels). (**c**) Direct bioprinting of pre-formed multicellular aggregates. Inset: a GFP-tagged nuclear transcription factor enables single-cell visualization. (**d**) After several days, the aggregates cultured only in growth media maintained their original spherical morphologies, while aggregates exposed to hTGF-β1 adopted invasive morphologies. This controllable invasion behavior implicates the chosen architecture and GelMA bioink as a relevant system for deeper functional studies (n = 1 gel for each condition). (**e**) An idealized model of intratumoral heterogeneity. By day 14, the separate cell populations grew to form a dense and seamless regional interface, likely suitable for longitudinal interrogation of emergent tumormosaicism (n = 4 gels).

The cells and matrix proteins of the tumor microenvironment are extremely heterogeneous, and aberrant spatial evolution of these discrete components is often associated with matrix invasion and subsequent metastasis^20^. To better understand these spatial transformations, we first sought to test our printer’s ability to photo-encapsulate dispersions of cancer cells from an established lineage (344SQ; murine lung adenocarcinoma^21^) within a multi-material core/shell architecture. Single cells were suspended at high density (20 × 10^6^ cells/mL) in a soft GelMA bioink (5 wt%, G’ ≈ 250 Pa) and printed as a thin cylindrical core (200 μm) fully encased on all sides by a larger acellular shell. (Fig. 5a). The importance of this acellular shell is subtle, yet critical. Traditional 3D assays use microwells, sheets, discs, and cubes to encapsulate cells throughout an entire volume, which allows highly migratory cells to escape the structure’s interior and undergo rapid 2D spreading along the structure’s exterior surfaces. We initially observed this phenomenon in the absence of an acellular shell, where escaped cells would rapidly coat the gel’s exterior and prevent optical interrogation of the underlying volume. Multi-material printing allowed us to circumvent this problem by constructing an acellular hydrogel shell around the entire cellular core, supporting longitudinal observation of 3D populations without the confounding presence of undesired 2D migration.

Immediately post-fabrication, we observed successful restriction of cells to their interior core domain, with no off-target encapsulation in the surrounding acellular shell (Fig. 5b). Gels were cultured in growth media for up to 5 days, at which point we observed apparent proliferation accompanied by multicellular aggregation into spherical structures. Autonomous aggregation is a well-known behavior of 344SQ cells when encapsulated in other biologically derived materials such as and collagen and Matrigel^22^, which suggests that our own printable GelMA bioink offers a potentially comparable environment for deeper biological study. Additionally, the appearance of growth and aggregation indicates that the acellular GelMA shell did not prevent diffusional access to oxygen and nutrients.

Because these 344SQ cancer cells proliferate so rapidly, multicellular aggregates were quickly encroached by neighboring aggregates, and by day 8 they had coalesced into a dense, unified structure. This morphology provided little space to evaluate the evolution of invasive cellular protrusions. Therefore, rather than printing dispersions of single-cells and waiting for autonomous aggregation, we moved to direct printing of spherical aggregates, pre-formed by culturing in hydrophilically passivated PDMS microwells^23^. The printing process was identical for both aggregates and single cells, apart from increasing layer heights from 200 to 300 µm to accommodate the larger size of the aggregates. Importantly, we found that printed aggregates maintained their tight spherical structures during fabrication and did not break apart into loose single cells (Fig. 5c).

To evaluate this system’s potential for studying tumor invasion, we introduced 5 ng/mL of hTGF-β1 growth factor to the encapsulated aggregates and monitored their behavior for several days in culture. While aggregates in normal growth media maintained their spherical morphologies, aggregates exposed to hTGF-β1 produced highly invasive morphologies as evidenced by the development of numerous cellular protrusions (Fig. 5d). This specific morphological response of 344SQ cells to hTGF-β1 has been well characterized in collagen^22^, Matrigel^24^, and bioactive PEGDA^24^, which further implicates our GelMA bioink as a potentially suitable material for mimicking aspects of the tumor microenvironment. Finally, we constructed a simplified model of intratumoral heterogeneity (Fig. 5e). Two sub-populations of 344SQ cells were suspended separately in soft GelMA bioinks (10 wt%, G’ ≈ 4000 Pa), and printed in a nested lattice architecture to mimic the organization of sub-clonal expansion in vivo^25,26^. The sub-populations of interest have distinct genetic modifications previously used to study metastatic progression^27,28^ however the specific details of these gene circuits are beyond the scope of this study. By day 14 the populations had grown to form a dense and seamless interface, which we expect to be useful for future explorations of emergent tumor mosaicism.

## Discussion

Mammalian anatomies comprise spatially heterogeneous arrangements of cells and extracellular components. In humans there exist hundreds of different cell types^29^ with unique genomic and proteomic profiles, all of which are physically and biochemically bound together by tissue-specific matrix proteins to perform complex functions. But the emergence of cooperative function between highly specialized tissue systems is fundamentally dependent on the physical separation of these components (e.g. parenchyma, stroma, vasculature) into restricted geometric configurations that both drive and limit physicochemical interactions. These architectures can also evolve temporally to modulate function, suggesting a need for tunable geometric design freedoms that are tissue- and application-specific. Simple co-culture assays have indeed provided enormous biological insight regarding heterotypic interactions, but these techniques often rely on homogenized mixtures of parenchymal and stromal cells^22,30–32^, which do not fully capture the native microenvironment’s discretized patterns, compartments, and multi-laminar structures.

To address this need for spatially controlled heterogeneity in vitro, here we explore not only the wide range of 3D architectures enabled by multi-material printing (multi-laminar stacks, nested co-planar features, vessel-containing structures) but also several different bioinks (PEGDA, MeHA, GelMA, photoclickable PEG)—which are known to have variable bioactivity and degradability^33–36^. While there is still much to be learned from gels with compositional uniformity, greater insights can surely be captured from access to multi-material architectures that integrate discrete cellular and extracellular components.

Towards multi-material in vitro tissue systems, multi-nozzle extrusion printing has clearly established itself as an effective means of heterogeneous construction and now exists in several commercial formats. However, due to the inherent difficulty of fusing cylindrical filaments into space-filling volumes during point-by-point scanning, extrusion is currently limited in its ability to fabricate seamless and void-free multi-material structures. With layer-by-layer manufacturing, MMSLA presents a void-free and higher resolution alternative. But MMSLA bioprinting with full 3D control has only been explored a handful of times in the literature^14,37,38^. Therefore, the full breadth of biological applications potentially unlocked by this technology has yet to be examined. Here, the simplest geometric format would be side-by-side co-culture patterning in a co-planar thin sheet gel, suitable for assays of interfacial phenomenon (e.g. osteochondral gradient formation, wound healing, tumor invasion). We therefore chose to demonstrate this architectural motif in Fig. 4 and show through morphometric image analysis that our MMSLA bioprinter faithfully produces 3D design files with extremely high accuracy and reproducibility. Importantly, we see that the interfacial contours between nested post/hole pairs form a tight fit without voids or seams, which would help ensure that cells migrate across regional domains without interruption. Attempts to build similar multi-material structures with extrusion bioprinting could yield interfacial artifacts due to incomplete fusion of adjacent cylindrical filaments.

Even more exciting is the opportunity to fabricate multi-material vascular structures with MMSLA. Our group has recently demonstrated that the use of biocompatible photoabsorbers in hydrogel SLA enables broad architectural control over vascular networks^12^. Here we extend this breakthrough to include systems with bulk heterogeneity (Fig. 3c) and vascular wall heterogeneity (Fig. 3d), which could serve as platforms to study the transmural behaviors of tumor cells^39^ or leukocytes^40^.

Towards fundamental biological assays, we imagine these previously unexplored architectures as platforms to study critical heterotypic interactions in the tumor microenvironment. Due to its known metastatic behavior, we chose to study the 344SQ cell line, which was originally derived from murine lung adenocarcinoma and exhibits genetic similarities to human lung adenocarcinoma^21^. Excitingly, we found that dispersed single cells and pre-formed multicellular aggregates were able to manifest well characterized phenotypic behaviors when printed in multi-material core/shell architectures. This design, with its internal cellular compartment, could be useful for studying how biological interfaces evolve as primary tumors begin to invade healthy stromal and parenchymal tissues. Furthermore, even individual tumors themselves exhibit spatial heterogeneity; while the majority of tumors originates from a single cell^41,42^, clonal expansion can yield genetic and phenotypic diversification that shapes the tumor’s anatomy and builds therapeutic resistance^43^. Accordingly, we conclude this work by devising an idealized model of intratumoral heterogeneity by spatially organizing phenotypically unique populations of tumor cells. Combining the two in heterogeneous mosaic architectures therefore presents a unique opportunity to study how sub-clonal populations cooperate to drive tumor growth^44^.

Together, these studies demonstrate the potential of MMSLA bioprinting to generate unique biological structures and assays, in which the influence of locally defined heterogeneity can be interrogated. We believe this work has provided clear evidence of the high geometric precision and diverse architectural freedom afforded by our MMSLA bioprinter. We expect these demonstrations will further validate MMSLA bioprinting as a powerful tool within the field of biofabrication, enabling deeper explorations of emergent behaviors and pathologies that occur at biological interfaces.

Moving forward, we acknowledge the limitations of our printing system with respect to scale, resolution, automation, and material selection . For projection stereolithography, feature size is limited by the physical dimensions of the projector’s pixels, which are usually on the order of tens of microns. This precludes fabrication of heterogeneous structures with sub-cellular resolution, which can otherwise be achieved using advanced laser-based technologies such as two photon polymerization^45^. Our printer is also limited with respect to scale, as large mosaics with complex internal heterogeneity—such as volumetric imaging datasets^46^—could require dozens of rinse steps that make this technique undesirable. However, these limitations of resolution and scale are largely application specific.

More generally, we also recognize that our printer does not exhibit the same level of full automation that other groups have engineered for MMSLA workflows. Despite the efficient synchronization between build plate, sled, and photomask projection sequence, our printing strategy requires the user to manually wash and dry the build plate between material selections. To reduce print times, human error, and the risk of microbial contamination, future iterations should incorporate an automated fluid exchanger. As previously described, these systems confine the build plate within a sealed fluidic cell that is filled and purged by elegant valve configurations to automate rinsing and material selection^16,17^. On the other hand, our manual rinsing strategy does not require a confined fluidic cell and thus provides more flexibility to create larger structures.

Lastly, despite our ability to integrate several synthetic and natural derived hydrogel bioinks, we are limited to materials with photocrosslinkable functional groups (e.g. acrylate, norbornene) and did not explore truly distinct classes of materials such as plastics, silicones, ceramics, or metals. Notably, MMSLA has previously been used to integrate acrylated hydrogels with acrylated plastics to build a semi-porous microfluidic chip^15^.

With full consideration of these strengths and limitations, we expect that our findings will not only validate MMSLA as a powerful bioprinting method, but also galvanize broad efforts within the scientific community to adopt and adapt the technology.

## Materials and methods

### Preparation of photosensitive materials

Poly(ethylene glycol) diacrylate (PEGDA; 3400 or 6000 Da) was prepared by reacting lyophilized poly(ethylene glycol) with triethylamine (TEA) and acryloyl chloride in anhydrous dichloromethane under argon as described previously^47^. Yields were typically in the range of 80–90% with acrylate substitution typically ≥ 85% as verified by ^1^H NMR integration at the characteristic peaks (6.4, 6.1, 5.8 ppm) of the PEG methylene protons adjacent to the acrylate. PEGDA stock solutions were made by dissolving powder at 50 wt% in PBS, followed by sterile filtration and then storage at − 20 or − 80 °C until use. When thawed, stocks were used within 3 days.

Gelatin methacrylate (GelMA) was synthesized as previously described^48^, with slight modifications. Methacrylic anhydride (1 mL per 10 g of gelatin) was added dropwise to gelatin (derived from porcine skin; type A; gel strength 300) dissolved at 10 wt% in a carbonate/bicarbonate buffer (30 mM sodium carbonate, 70 mM sodium bicarbonate; pH ≈ 9.75) at 50–55 C for 4 h, followed by precipitation in 190 proof ethanol (1:20 volume ratio). The precipitate was allowed to dry for several days at ambient conditions, followed by overnight 60 C dissolution at 20 wt% in PBS. The resultant stock solutions were noticeably turbid, likely due to unwanted self-polymerization of the methacrylic anhydride, and therefore filtered using 0.45 μm filters (polyethersulfone) to remove larger particulates. The product was then sterilized using 0.2 μm filters and stored at − 20 or − 80 °C until use. When thawed, stocks were used within 24 h. Substitution was typically in the range of 40–60% as verified by ^1^H NMR using a previously described protocol^49^.

Lithium phenyl-2,4,6-trimethylbenzoylphosphinate (LAP) was synthesized as previously described^50^ by reacting an equimolar ratio of dimethyl phenylphosphinite with 2,3,6-trimethylbenzoyl chloride under argon overnight followed by addition of 4 molar excess lithium bromide dissolved in 2-butanone. The reaction mixture was heated to 50 °C to allow formation of a solid precipitate (≈ 10–30 min) and then cooled to room temperature for 4 h. Using a Buchner funnel, the resulting slurry was rinsed with excess volumes of 2-butanone and diethyl ether, and the resulting precipitate was allowed to dry for several days before storing under nitrogen at 4 °C until use. For stock solutions, powder was dissolved at 200 mM in PBS, sterile filtered, protected from light, and used within 3 weeks.

Photoclickable PEG reagents were purchased from a commercial supplier (PEG-dithiol, 3400 Da; 8-arm PEG norbornene, 20 kDa; JenKem Technology, Plano, TX). Stock solutions for each component were made by dissolving powder at 25 wt% in PBS and used within 24 h. To formulate print solutions, PEG-dithiol and 8-arm PEG norbornene were used together at 10 wt% in an equimolar ratio (4.12 and 5.88 wt%, respectively) to ensure equal concentrations of thiol and norbornene functional groups (23.5 mM each). Methacrylated hyaluronic acid was kindly gifted from Dr. Sanjay Kumar's lab (Dept. Bioengineering, U.C. Berkeley), and used at 5 wt%.

For glass methacrylation, standard microscope slides were first cleaned by sonication (1 h, 37 C) in a MilliQ water solution with 1.5 wt% Alconox powdered detergent (Alconox, White Plains, NY). The glass slides were then thoroughly rinsed in excess MilliQ water, followed by rinsing in excess ethanol (190 proof). Slides were transferred to a slinanizing solution of 300 mL ethanol (190 proof), 15 mL of silane compound (3-trimethoxysilyl-propyl-methacrylate; Sigma-Aldrich, St. Louis, MO), and 10 mL dilute acetic acid (1 mL glacial acetic acid, 10 mL MilliQ water). Slides were allowed to react for 12–24 h, followed by rinsing in excess ethanol (190 proof) and then excess MilliQ water. Slides were then baked at 60 C for 5–12 h, and stored for up to 2 months protected from light at 4 °C.

### Bioprinter development

Our MMSLA bioprinter combines a custom printing apparatus and a commercially available projection system. The printing apparatus is made from 4 major components that are attached to a Misumi aluminum frame (Misumi USA, Schaumburg, Illinois): (1) RepRap Arduino Mega Board (RAMBo; UltiMachine, South Pittsburg, TN) microcontroller for motor control; (2) a build plate that moves the hydrogel up and down during printing to address individual layers; (3) a silicone sled (glass-coated PDMS; Sylgard 184, Dow Corning, Midland, MI); and (4) a base with a 45° mirror to reflect the projected images up through the silicone sled to irradiate the bioinks. The silicone sled, which holds the bioink droplets, is translated by a pulley to provide automated material selection. The sled also contains a rinsing station, where a mounted petri dish collects the falling saline rinsing fluid as it drips off the actively printing object. The saline solution consisted of dilute photopolymer (2.5–10 wt%) in PBS. The printer body, mirror mount, microcontroller housing, silicone sled, and build plate were 3D printed with an Ultimaker 2 (Ultimaker, Netherlands) in food-grade poly(lactic acid) (PLA) plastic filament (Ultimachine).

The projection device consists of either a commercially available Acer H6510BD projector (Acer, San Jose, CA), with a display resolution of 1920 × 1080 pixels and a light intensity of 3000 Lumens, or a PRO4500 optical Engine (Wintech, Carlsbad, CA), with a display resolution of 1280 × 800 pixels containing a 405 nm LED, attached to a computer used for projection of photomasks and motor control. The projector was placed in front of the printing apparatus and focused to obtain an XY-resolution of 38 µm (Acer) or 50 µm (PRO4500). No modification to the projectors were made. Power output from the projectors was measured with a Model 308 handheld light intensity meter (OAI Instruments, San Jose, CA) with either a 540 nm probe (Acer) or a 400 nm probe (PRO4500).

The commercial availability of all non-custom components of this bioprinter will enable other groups to easily adopt this system for fabrication of multi-layered hydrogels with multiple materials. A GitHub repository (https://github.com/MillerLabFTW/MMSLA) contains design files for the 3D printed components and firmware of our developed printing system.

### Multi-material hydrogel stereolithography

To demonstrate the utility of this technology in fabrication of multi-material hydrogels, arbitrary geometries were modeled in SolidWorks (Dassault Systemes, Waltham, Massachusetts) or Blender (Blender Foundation, Amsterdam, Netherlands). For fabrication of hydrogels containing multiple materials within the same plane, 3D models containing three rectangular 100 µm thick base layers and an additional 100 µm thick layer consisting of various patterns. Before printing, the projector focus was adjusted as well as the brightness to result in light intensity of 40 mW/cm^2^ when measured with a 540 nm probe (for the Acer projector) or 16.5 mW/cm^2^ when measured with a 400 nm probe (for the PRO4500 projector).

For initial acellular prints (Figs. 2, 3, 4), bioinks were composed of 15–20 wt% PEGDA (3.4 kDa or 6 kDa) with 17–34 mM LAP. For visualization and quantification, various fluorescent tracers were incorporated into the bioink formulation: blue, red, or green fluorescent beads (1–10 μm; Magsphere Inc., Pasadena, CA), and fluorescent dextrans (150 kDa FITC-dextran, or 70 kDa rhodamine dextran; Sigma Aldrich, St. Louis, MO). To print hydrogels with multiple materials, 100–1000 µL bioink droplets were pipetted onto the silicone sled with ≥ 25 mm spacing in between each droplet. The printing process for a structure with 3 base layers and a feature layer with 2 different materials was as follows: (1) the build plate is lowered onto the far left droplet which does not contain any dyes and printing of 3 base layers is performed; (2) after the 3rd layer is patterned, the build plate withdraws from the droplet, the sled moves laterally to the second droplet (containing a fluorescent tracer), the build plate is lowered to the next layer height, and the projector projects the subsequent grayscale photomask consisting of an arbitrary geometry; (3) after the layer undergoes complete gelation, the projection turns off and the build plate withdraws from the droplet. The sled moves linearly until the build plate is above the rinsing station, the gel is then rinsed with excess saline solution to remove residual material; (4) the sled moves laterally to the position of the third droplet (containing a different colored fluorescent tracer) and another pattern is projected after the build plate lowers to the same position as the previous layer. After completion of printing, the hydrogel is removed from the glass build plate and extensively rinsed with deionized water or PBS to remove unreacted components before moving to a multi-well plate for imaging or culture. Our current system supports integration of up to 4 materials in a single print, and it is likely that the use of a longer silicone sled would support an even greater number of materials.

In addition to PEGDA chemistry, we also demonstrate the printing of multi-material hydrogel structures using methacrylated hyaluronic acid (5 wt% MeHA), gelatin methacrylate (5 wt% GelMA), and photoclickable PEG. The photoclickable PEG gels were formulated as an equimolar mixture of 8-arm PEG-norbornene (5.88 wt%, 20 kDa) and PEG-dithiol (4.12 wt%, 3.5 kDa).

### Morphometric image analysis of multi-material print fidelity

To quantify the geometric fidelity of printed structures, we designed and printed PEGDA hydrogels (20 wt%, 6 kDa) in a checkerboard pattern with fluorescent dextrans encapsulated for visualization (150 kDa FITC dextran; 70 kDa rhodamine dextran). The gel was designed with a 300 μm non-fluorescent base layer with a subsequent 100 μm feature layer consisting of 500 μm cylindrical posts nested within an open lattice. We wrote a custom image processing script in MATLAB (MathWorks, Natick Massachusetts) to measure the diameter size, centroid distance between adjacent features, spatial alignment of structures, and circularity of posts and holes. The diameter size and centroidal distance from nearest neighbors were measured using the blob analysis tool in MATLAB; spatial alignment was measured as distance from hole centroid to post centroid, and circularity was defined by the following equation:

$$circularity = \;\frac{4\pi * area}{{perimete{r^2}}}$$

### Bioprinting single cells and multicellular aggregates

For all bioprinting experiments, cells or aggregates were suspended in bioinks of 5 or 10 wt% GelMA and 10 mM LAP (in PBS) with projection intensities of 10 mW/cm^2^. For Fig. 5B/E cells were used at a density of 20 × 10^6^ cells/mL. Print layers were 200 μm thick and photocrosslinked for 60 s. For Fig. 5C/D, multicellular aggregates of 344SQ were pre-formed in hydrophilically passivated PDMS microwells^23^, using 50 cells/aggregate and printing with 50,000 aggregates/mL. Here, print layers were increased to 300 μm to accommodate the larger size of the aggregates, and thus required longer exposure times of 120 s to fully crosslink the entire layer’s depth. Rather than scraping the gels off the build plate’s glass substrate, the glass itself was simply peeled off the build plate, which was held on by thin double-sided tape. The glass and gel were then transferred together to a 6-well plate, where they were cultured and imaged for up to 15 days. The covalent bonds between the gel and methacrylated glass allowed the gels to remain fully attached during the entire culture period. Without methacrylation the gels would detach after a 2–4 days and float in the media, making it difficult to image.

### Cell culture

The lung adenocarcinoma cell line 344SQ—originally isolated from murine tumors and continuously maintained in house—were cultured at 37 °C, 5% CO_2_ in RPMI 1640 (Corning Inc, Corning NY) supplemented with 10% fetal bovine serum (Atlanta Biologicals, Flowery Branch, GA) and 1× penicillin–streptomycin (Gibco Life Technologies, Grand Island, NY, final concentrations of 100 units/mL and 100 μg/mL, respectively). Cells were passaged at confluences of 60–90% w/0.25% Trypsin/EDTA (Caisson Labs, East Smithfield, UT). Multicellular aggregates of 344SQ cells were prepared as previously described^23^. Briefly, PDMS microwell arrays were fabricated using ultrahigh-throughput laser ablation, followed by sonication in isopropanol to remove leftover debris. The microwell arrays were then passivated against cell adhesion by incubating in Pluronic F-127 (5 wt% in PBS, Sigma-Aldrich, St. Louis, MO). After trypsinization, cells were pipetted into the microwells at concentrations calculated to yield 50 aggregates per microwell. After 18–24 h of culture, aggregates were dislodged from their microwells by manual agitation (i.e. repeated aspiration/expulsion) with a standard 200 or 1000 μL pipette. Aggregates were suspended in the 5 wt% GelMA bioink immediately before printing. In Fig. 5C, individual 344SQ cells were visualized using a GFP-tagged version of the Zeb1 nuclear transcription factor, which required genetic modification of the 344SQ cells^28,51^. This fusion protein was not constitutively expressed, but rather induced using 2 μg/mL doxycycline.

### Microscopy

Brightfield and fluorescent images were obtained on a Nikon Eclipse Ti inverted epifluorescent microscope (Nikon Instruments Inc., Melville, NY) equipped with a Zyla 4.2 sCMOS camera (Andor, South Windsor, CT).

## Data Availability

All data and design files are located at https://github.com/MillerLabFTW/MMSLA and are completely open source under the Creative Commons BY license (CC BY 4.0); free to copy, distribute, remix, transform, and build upon for any purpose.

## Abbreviations

SLA: Stereolithography
MMSLA: Multi-material stereolithography
PDMS: Poly(dimethyl siloxane)
PEG: Poly(ethylene glycol)
PEGDA: Poly(ethylene glycol) diacrylate
GelMA: Gelatin methacrylate
MeHA: Methacrylated hyaluronic acid
LAP: Lithium phenyl-2,4,6-trimethylbenzoylphosphinate
kDa: Kilodalton
344SQ: Murine lung adenocarcinoma cell line
hTGF-β1: Human transforming growth factor β1
